# 276 Structure and Leadership of Inpatient Antibiotic Stewardship Programs: Findings from the APIC MegaSurvey 2025

**DOI:** 10.1017/ash.2026.10638

**Published:** 2026-06-23

**Authors:** Amy Spallone, Guy Handley, Micah Bhatti

**Affiliations:** 1 MD Anderson Cancer Center

## Abstract

**Background:** Candida kefyr, a non-albicans-yeast species, is recognized as a potential emerging pathogen among immunocompromised patients. Previous epidemiological studies have indicated that hematologic malignancy is a key risk factor for invasive infection among patients with cancer and those with neutropenia; however, the clinical impact is not well defined. **Methods:** As part of a clinical inquiry, we conducted a retrospective review of laboratory-confirmed cases of C. kefyr to understand its occurrence and distribution at an NCI-designated comprehensive cancer center from January 2024 through September 2025. Demographic, laboratory, and clinical outcome data were extracted from the electronic medical record. Epidemiologic data were reviewed to evaluate for potential trends or characteristics of patients with C. kefyr infection or colonization **Results:** Overall, 24 isolates were identified from 21 distinct patients. The majority were from urinary sources (71.4%). The majority of cases were considered colonizers (61.9%), but eight (38.1%) patients had clinical infections, including two (25%) bloodstream infections. The majority of patients were female (15, 71.4%) with a median age of 70 years old and an Eastern Cooperative Oncology Group (ECOG) score of 3 or greater (13, 62%). In contrast to published literature, a majority (17, 81.0%) of patients had solid tumors, and hematologic malignancy was less common, with only one patient having severe neutropenia at the time of culture. The majority (16, 76.2%) of patients had some form of central venous catheter. Only two (9.5%) patients were receiving antifungal therapy at the time of culture, with one additional patient having received antifungal therapy within the preceding 90 days. Overall, the 90-day mortality in patients with C. kefyr isolated was high (14/21; 66.7%), though not necessarily related to the organism. **Conclusions:** C. kefyr is a potential emerging pathogen, and patients with colonization or infection were noted to have substantial 90-day mortality. In contrast to prior studies, we observed several cases in non-neutropenic patients with solid tumors. Additional comparative studies with other organisms or Candida species are needed to better characterize risk factors, clinical impact, and the role of this organism in cancer patients.

